# Analytic performance studies and clinical reproducibility of a real-time PCR assay for the detection of epidermal growth factor receptor gene mutations in formalin-fixed paraffin-embedded tissue specimens of non-small cell lung cancer

**DOI:** 10.1186/1471-2407-13-210

**Published:** 2013-04-27

**Authors:** Patrick O’Donnell, Jane Ferguson, Johnny Shyu, Robert Current, Taraneh Rehage, Julie Tsai, Mari Christensen, Ha Bich Tran, Sean Shih-Chang Chien, Felice Shieh, Wen Wei, H Jeffrey Lawrence, Lin Wu, Robert Schilling, Kenneth Bloom, Warren Maltzman, Steven Anderson, Stephen Soviero

**Affiliations:** 1 Roche Molecular Systems, Inc., 4300 Hacienda Blvd, Pleasanton, CA, 94588, USA; 2 GE Healthcare/Clarient Diagnostic Services, Inc., Aliso Viejo, CA, USA; 3 Quintiles Laboratories, Westmont, IL, USA; 4 Laboratory Corporation of America, Research Triangle Park, NC, USA

**Keywords:** EGFR mutation testing, Molecular diagnostics, Companion diagnostics, Non-small cell lung cancer, Analytical validation, Reproducibility

## Abstract

**Background:**

Epidermal growth factor receptor (EGFR) gene mutations identify patients with non-small cell lung cancer (NSCLC) who have a high likelihood of benefiting from treatment with anti-EGFR tyrosine kinase inhibitors. Sanger sequencing is widely used for mutation detection but can be technically challenging, resulting in longer turn-around-time, with limited sensitivity for low levels of mutations. This manuscript details the technical performance verification studies and external clinical reproducibility studies of the cobas EGFR Mutation Test, a rapid multiplex real-time PCR assay designed to detect 41 mutations in exons 18, 19, 20 and 21.

**Methods:**

The assay’s limit of detection was determined using 25 formalin-fixed paraffin-embedded tissue (FFPET)-derived and plasmid DNA blends. Assay performance for a panel of 201 specimens was compared against Sanger sequencing with resolution of discordant specimens by quantitative massively parallel pyrosequencing (MPP). Internal and external reproducibility was assessed using specimens tested in duplicate by different operators, using different reagent lots, instruments and at different sites. The effects on the performance of the cobas EGFR test of endogenous substances and nine therapeutic drugs were evaluated in ten FFPET specimens. Other tests included an evaluation of the effects of necrosis, micro-organisms and homologous DNA sequences on assay performance, and the inclusivity of the assay for less frequent mutations.

**Results:**

A >95% hit rate was obtained in blends with >5% mutant alleles, as determined by MPP analysis, at a total DNA input of 150 ng. The overall percent agreement between Sanger sequencing and the cobas test was 96.7% (negative percent agreement 97.5%; positive percent agreement 95.8%). Assay repeatability was 98% when tested with two operators, instruments, and reagent lots. In the external reproducibility study, the agreement was > 99% across all sites, all operators and all reagent lots for 11/12 tumors tested. Test performance was not compromised by endogenous substances, therapeutic drugs, necrosis up to 85%, and common micro-organisms. All of the assessed less common mutations except one (exon 19 deletion mutation 2236_2248 > AGAC) were detected at a similar DNA input level as that for the corresponding predominant mutation.

**Conclusion:**

The cobas EGFR Mutation Test is a sensitive, accurate, rapid, and reproducible assay.

## Background

Lung cancer has the highest incidence of all solid organ cancers and is the most common cause of death from cancer worldwide, accounting for over 1.6 million new cases annually and 1.38 million deaths [1]. Almost 85% of all lung cancers are non-small cell lung cancer (NSCLC). The observation that the epidermal growth factor receptor (EGFR) is over-expressed in most cases of NSCLC led to the development of the specific anti-EGFR tyrosine kinase inhibitors (TKIs) gefitinib and erlotinib as targeted therapeutic agents. However, clinical trials with these agents revealed that in most cases, responders harbored specific activating mutations in exons 18–21 which collectively encode the kinase domain of the *EGFR* gene [2-5]. The majority of mutations that have been associated with sensitivity to gefitinib and erlotinib are located in exon 19 (45%) and exon 21 (40–45%), although ~5% are located in exon 18 and <1% in exon 20 [6]. In addition, certain mutations in exon 20, such as T790M, predict resistance to these TKIs [7].

The association between sensitizing mutations in the *EGFR* gene and response to treatment has led to recommendations by major oncology organizations that NSCLC tumors should be tested for the presence of these mutations before treatment [8-10]. Thus, from a practical perspective, optimal care of patients will depend on interactions between a patient’s pulmonologist and oncologist, relying on information from the molecular pathology of the tumor tissue [11]. In recognition of the need for accurate testing, these organizations have started consolidating guidelines for molecular testing in lung cancer to follow standards for sensitivity, specificity, and time to results to ensure quality of patient treatment [12].

As with other tumor types, diagnostic assays should be optimized for use with formalin-fixed paraffin-embedded tissue (FFPET) specimens, which continue to represent the vast majority of NSCLC samples in clinical practice today. Molecular testing in NSCLC poses particular challenges for the pathologist and clinician alike. In many cases the amount of tumor tissue available for testing (e.g. bronchial biopsy) is very limited and, given the growing number of molecular and immunohistochemical studies that are performed as part of the diagnostic workup, there are competing diagnostic demands for the small amount of available material. Thus, an optimal diagnostic test should require a small amount of DNA. Furthermore, many patients with metastatic NSCLC are often quite ill and require prompt initiation of targeted therapy when indicated, making a rapid molecular assay highly desirable.

The importance of using standardized techniques for both extraction and molecular analysis was stressed by a recently convened expert working group who discussed the challenges of NSCLC diagnosis in the current era [11]. This group recommended against using laboratory developed tests, as such methods are subject to great inter- and intra-laboratory variability and do not always pass adequate quality control schemes that ensure reproducibility of results. Instead, the group recommends, where possible, using certified diagnostic kits with prior laboratory validation.

We designed a highly sensitive, specific, reproducible test that detects mutations in exons 18, 19, 20, and 21 in tumor samples from patients with NSCLC to identify individuals who are most likely to respond to EGFR TKI therapy using one 5-micron tissue section. Here, we present the technical performance verification studies of the cobas EGFR Mutation Test, including studies of the analytic sensitivity, internal and external reproducibility, minimal tumor content, interfering substances, effects of necrosis, and cross-reactivity with other mutations.

## Methods

### Materials

FFPET specimens of NSCLC tumors were obtained from US and European commercial vendors: Analytical Biological Services Inc. (Wilmington, DE, USA), Asterand, Inc. (Detroit, MI, USA), BioServe (Beltsville, MD, USA), Conversant (Huntsville, AL, USA), Cureline Inc. (South San Francisco, CA, USA), Cytomyx (Lexington, MA, USA), Discovery Life Sciences, Inc. (Los Osos, CA, USA), ILSBio, LLC (Chestertown, MD, USA), Indivumed (Hamburg, Germany), OriGene (Rockville, MD, USA), and ProteoGenex (Culver City, CA, USA). In addition, FFPET specimens of NSCLC tumors were provided by Astellas Pharma US, Inc. (Deerfield, IL, USA). All specimens were aged between 3 and 10 years.

Human epidermal growth factor receptor (HER) plasmids: HER2, HER3, and HER4 were purchased from Integrated DNA Technologies (San Diego, CA, USA).

K562, human genomic DNA, used as wild-type sequence, was obtained from the human lymphoma cell line K562 (Promega, Madison WI; part number DD201X).

### Ethics statement

Methods described below were not used in the diagnosis or treatment of any patients. Patient consent forms were obtained through the commercial vendor. RMS and the principal investigators from the external reproducibility study abided by the International Conference on Harmonisation Good Clinical Practice Guidelines and regulations of the US Food and Drug Administration (FDA) in the conduct of this study. Before the start of the study, the protocol and other documents necessary for participating sites to perform the study was submitted to an independent Institutional Review Board (IRB) in accordance with FDA and local legal requirements. IRB approval was obtained at each site.

### cobas EGFR mutation test

**Figure 1:**
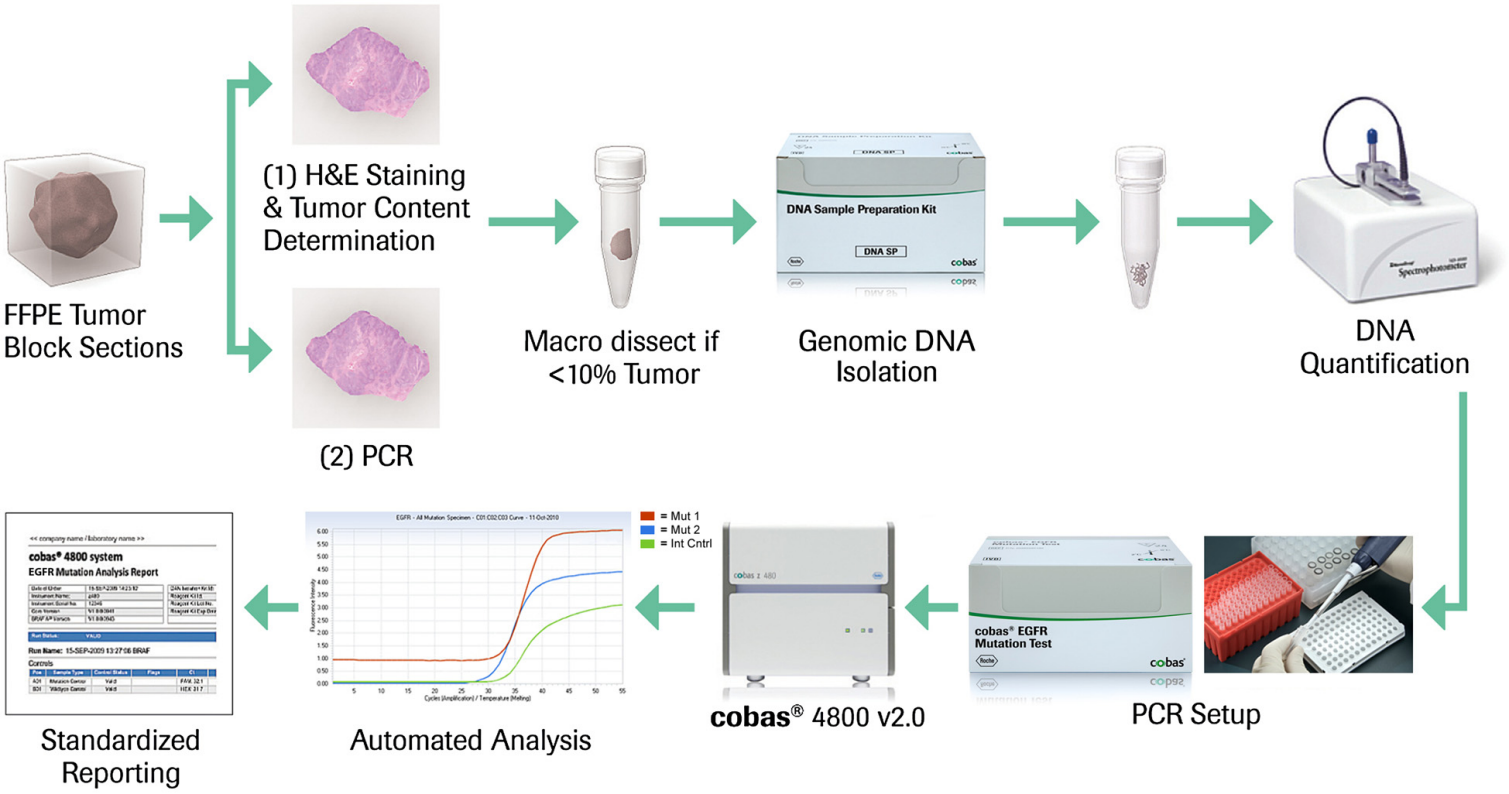
**cobas EGFR Mutation Test workflow.** EGFR, epidermal growth factor receptor; FFPE, formalin-fixed paraffin-embedded; H&E, hematoxylin and eosin; PCR, polymerase chain reaction.

The cobas EGFR Mutation Test (“cobas EGFR test”, Roche Molecular Systems, Inc, Branchburg, NJ, USA) is a CE-IVD marked multiplex assay that uses allele-specific polymerase chain reaction (AS-PCR) to detect 41 mutations in exons 18, 19, 20, and 21 in FFPET specimens of human NSCLC. The test consists of two major steps: (1) a manual DNA isolation step, and (2) PCR amplification and detection of target DNA using complementary primer pairs and oligonucleotide probes labeled with fluorescent dyes (Figure 1). The test is designed to detect G719X (G719A, G719C, and G719S) in exon 18; deletions and complex mutations in exon 19; S768I, T790M, and insertions in exon 20; and L858R in exon 21. The specific mutations detected by the assay are detailed in Table 1. A mutant control and a negative control are included in each run to confirm the validity of the run. The test uses 150 ng total input DNA, an amount which can typically be extracted from a single 5 μm section of an FFPET specimen using the cobas DNA Sample Preparation Kit (Roche Molecular Systems, Inc, Branchburg, NJ, USA), following the standard package insert protocol [13]. Macrodissection of the tissue section is only required if the estimated tumor content is < 10% by pathological assessment. Mutation analysis is performed through real-time PCR analysis using the cobas 4800 System (version 2.0), and the analysis of raw data and reporting of results are fully automated. The DNA isolation, amplification and detection, and result reporting can be performed in less than 8 hours. Testing for this study was conducted with cobas 4800 System Software (version 2.0.0.1028). Analysis was performed with the EGFR Analysis Package Software V.1.0

**Table 1:** cobas EGFR mutation test coverage

**Exon**	**Mutations detected**	**Mutation report call**
**18**	G719A, G719C, G719S	G719X
**19**	29 deletions	Exon 19 deletion
**20**	T790M	T790M
	S768I	S768I
	5 insertions	Exon 20 insertion
**21**	L858R (2 variants*)	L858R

EGFR, epidermal growth factor receptor; PCR, polymerase chain reaction.

*2573 T > G; 2573_2574TG > GT.

### Sanger sequencing

DNA from FFPET specimens using the same extraction method as described for the cobas EGFR test were extracted and amplified at Roche Molecular Systems, and sent out for 2× bidirectional Sanger sequencing (Sanger) by a Clinical Laboratory Improvement Amendments (CLIA)-certified laboratory (SeqWright, Houston, TX, USA) using a validated protocol.

### Quantitative massively parallel pyrosequencing

NSCLC FFPET-derived DNA blends and specimens that gave discordant cobas EGFR test and Sanger test results as well as a randomly selected subset of concordant specimens were tested using a quantitative massively parallel pyrosequencing method (“MPP”, 454 GS Titanium, 454 Life Sciences, Branford, CT, USA) [14]. DNA was extracted and amplified at Roche Molecular Systems prior to being sent to a CLIA-certified laboratory (SeqWright, Houston, TX, USA) to be sequenced using a validated protocol for EGFR mutation detection. The analytical sensitivity of MPP for EGFR mutations was validated to a limit of detection of 1.25%.

### Analytical sensitivity

The analytical sensitivity of the cobas EGFR test was assessed using FFPET-derived DNA blends and plasmid DNA blends. For the FFPET DNA blends, seventeen NSCLC FFPET specimens were selected for their mutation status (three positive for exon 19 deletions; three positive for L858R mutations; one positive for L858R and T790M mutations; one positive for S768I and G719C mutations; one positive for G719A mutation; one positive for an exon 20 insertion mutation; and seven *EGFR* wild-type specimens) as determined by Sanger sequencing. Specimen blends were prepared targeting approximately 10%, 5%, 2.5%, and 1.25% mutant DNA as quantified by MPP pyrosequencing. Serial dilutions of each specimen were prepared and eight replicates were tested with three cobas EGFR test reagent lots, yielding a total of 24 replicates per panel member.

Six plasmid constructs containing the most frequently observed mutation for each mutation group detected by the test were blended with K562 wild-type DNA such that each sample contained a ~5% blend of mutant plasmid at the copy number equivalent of 50 ng/PCR. Serial dilutions of each specimen were prepared to make panels with five members. DNA samples were diluted while leaving the percent mutation constant. An additional panel member containing 100% wild-type DNA was included to each panel. Each of the six levels of the six plasmid DNA blend specimens was tested with each of three unique cobas EGFR test lots. Three dilutions were formulated for each plasmid in each mutation group for each of the three reagent kit lots. Eight replicates of each of the three dilution series was tested for each of the three kit lots, yielding a total of seventy-two replicates per panel member.

### Method correlation

Analytical performance of the cobas EGFR test was compared against 2× bidirectional Sanger sequencing using 201 FFPET human NSCLC specimens. Correlation between the two methods was assessed by agreement analysis, including positive percent agreement (PPA), negative percent agreement (NPA), and overall percent agreement (OPA). Specimens with invalid results on either method were excluded from the correlation analysis. The cobas EGFR test results were considered invalid if any or all of the mutation calls were reported as invalid. Sanger sequencing results were considered invalid if any or all of the four exons failed to provide a valid result for a specimen. Sanger sequencing results were considered invalid if any or all of the four exons failed to provide a valid result for a specimen. Specimens with discordant cobas EGFR test and Sanger sequencing results and a randomly selected subset of specimens with concordant results were subjected to MPP.

### Repeatability/Reproducibility

The internal repeatability of the cobas EGFR test was evaluated using six NSCLC FFPET specimens: two *EGFR* wild-type and four *EGFR*-mutation positive specimens (one exon 19 deletion, one with G719X and S768I mutations, one with L858R and T790M mutations, and one with exon 20 insertion mutation). Testing was performed in duplicate by two operators, using two different reagent lots and two cobas z 480 analyzers over 4 days. Each operator performed one run per reagent lot per day for 4 days, giving a total of 16 runs and 32 replicate results for each of the six specimens.

The external reproducibility of the cobas EGFR test across three clinical laboratories using three reagent lots and two operators per site was evaluated using a 13-member panel of NSCLC specimens containing five different deletions in exon 19 and exon 21 L858R in the EGFR gene (Table 2). Operators performed blinded runs (two replicates of each panel member/run), on five nonconsecutive days, using a single instrument per site. A total of 180 DNA replicate specimens were prepared for each panel member, and each of the three sites performed a total of 780 tests. All statistical analyses were performed using SAS®/STAT® software. The sample identification numbers of panel members were randomized using PROC PLAN in SAS, and the order of panel members within each run was randomized. Only valid tests from valid runs were included in the statistical analyses.

**Table 2:** External reproducibility panel design

**Panel member**	**Mutation status**
1	Wild Type
2	Exon 19 – deletion mutation #1 – LOD
	(EX19_ 2235_2249del15 - 5% Mutation)
3	Exon 19 – deletion mutation #2 – LOD
	(EX19_2236_2250del15 - 5% Mutation)
4	Exon 19 – deletion mutation #3 - LOD
	(EX19_2239_2248 > C - 5% Mutation)
5	Exon 19 – deletion mutation #4 - LOD
	(EX19_2240_2254del15 - 5% Mutation)
6	Exon 19 – deletion mutation #5 - LOD
	(EX19_2240_2257del18 - 5% Mutation)
7	Exon 21 L858R mutation – LOD
	(EX21_ 2573T > G = L858R - 5% Mutation)
8	Exon 19 – deletion mutation #1 – 2 × LOD
	(EX19_ 2235_2249del15 - ≤10% Mutation)
9	Exon 19 – deletion mutation #2 – 2 × LOD
	(EX19_2236_2250del15 - ≤10% Mutation)
10	Exon 19 – deletion mutation #3 – 2 × LOD
	(EX19_2239_2248 > C - ≤10% Mutation)
11	Exon 19 – deletion mutation #4 – 2 × LOD
	(EX19_2240_2254del15 - ≤10% Mutation)
12	Exon 19 – deletion mutation #5 – 2 × LOD
	(EX19_2240_2257del18 - ≤10% Mutation)
13	Exon 21– L858R mutation – 2 × LOD
	(EX21_ 2573T > G = L858R - ≤10% Mutation)

The mutation status and percent mutant alleles of the specimens chosen for the repeatability and reproducibility studies was determined using Sanger sequencing and MPP, respectively, and tumor content was estimated for each specimen by assessment from an external pathologist. DNA was extracted and blended to create samples containing levels of EGFR mutation above the limit of detection for the cobas platform. The percentage of mutant alleles in the blended samples was verified by MPP.

### Potential interfering substances

The effects on the performance of the cobas EGFR test from two endogenous substances (hemoglobin and triglycerides) and nine therapeutic drugs that may be present in human NSCLC specimens (albuterol, ipratropium, fluticasone, ceftazidime, imipenem, piperacillin-tazobactam, cilastatin sodium, povidone iodide, and lidocaine) were investigated with 10 NSCLC FFPET specimens. Specimens were selected for mutation status based on Sanger and/or MPP. Five specimens were *EGFR* mutation-positive and five were wild type. Specimens were tested in the absence and presence of each potential interferent. Each potential interferent was spiked during the lysis step. Hemoglobin and triglycerides were added to achieve 1× the upper limit of normal concentration seen in common pathological conditions (as defined by the Clinical and Laboratory Standards Institute [CLSI] EP7-A2 Guideline; 2 g/L hemoglobin and 37 mM triglycerides) [15]. The therapeutic drugs were added to achieve a final concentration of 3× the maximal plasma concentration (as defined by the CLSI EP7-A2 Guideline) [15], if known. Povidone iodide was tested as a 10% weight by volume solution; lidocaine was tested at a concentration of 12 μg/mL, as recommended by the CLSI EP7-A2 Guideline [15].

### Effects of necrosis

The impact of tissue necrosis on the cobas EGFR test detection of mutations was evaluated. Twenty NSCLC FFPET specimens were tested in duplicate: ten specimens covering a range of percent mutation from the exon 19 deletion, S768I, L858R, G719X, and exon 20 insertion mutation groups, and ten wild-type specimens. Percent necrosis, as assessed by a pathologist, varied from 0% to 60% for mutant specimens and from 5% to 85% for wild-type specimens.

### Cross-reactivity

To confirm that other gene sequences homologous to the targeted *EGFR* exons do not interfere with the performance of the cobas EGFR test, potential cross-reactivity was assessed for three members of the ErbB family of receptor tyrosine kinases (HER2, HER3, and HER4). The homologous sequences in HER2, HER3, and HER4 corresponding to the probe-targeted portions of exons 18, 19, 20, and 21 in the *EGFR* gene were individually cloned into 12 plasmids (four exon regions per *HER* gene) and evaluated with the cobas EGFR test. We also sought to determine if the assay, which is designed to detect 29 deletions in exon 19 would also detect the rare exon 19 L747S point mutation, using a plasmid containing this mutation. Ten NSCLC FFPET specimens (four with EGFR mutations, six wild type) were evaluated in the presence (spiked to a concentration of 15,850 copies/PCR well, the equivalent of 50 ng of genomic DNA) and absence of each of the HER plasmids as well as the plasmid containing the L747S mutation. The plasmids were spiked into individual replicates of each of the ten specimens after extraction; one replicate of each of the 10 specimens was not spiked with plasmid and was used as the control.

### Genotype inclusivity

To assess the inclusivity of the assay for mutations in all four key exons of EGFR (exons 18–21), the detection of less common non-predominant *EGFR* mutations was studied for each of the four exons (G719X point mutations in exon 18, deletions in exon 19 deletions, insertions in exon 20, and a two base pair mutation that yields variant in the L858R mutation in exon 21). Plasmid constructs containing these less common mutations were blended with wild-type DNA (K562). The initial plasmid DNA input level was determined by the findings from the analytical sensitivity study for the predominant mutation (as detailed above). If the hit rate at this level was too low, then the next highest DNA input level was tested, with levels subsequently increased up a maximum of 50 ng/PCR. Each plasmid DNA blend sample was tested with one test kit lot, and a total of 24 replicates were tested per sample.

### Microorganism exclusivity

Ten NSCLC FFPET specimens (five mutation positive, five wild-type) were tested with two common respiratory microorganisms (*Haemophilus influenzae and Streptococcus pneumoniae*), Controls (normal substance level which did not contain any added organism) were used for all specimens. Microorganisms were spiked at 1e6 CFU/mL. A total of 30 test conditions were run.

## Results

### Analytical sensitivity

The analytical sensitivity of the cobas EGFR test for exon 19 deletion, L858R, S768I, T790M, G719X, and exon 20 insertion mutations was assessed using NSCLC FFPET-derived DNA blends and six plasmid DNA blends. For the FFPET-derived DNA blends the lowest percent mutation level that was associated with ≥95% hit rate with 50 ng/PCR reaction ranged from 1.3% to 5.6% (Table 3). For the plasmid blends, the amount of DNA in 5% copy equivalent to achieve ≥ 95% mutation detected rate ranged from 0.78 and 3.13 ng/PCR reaction (Table 4). Together, the data show that the cobas EGFR test can detect the predominant mutation for each of the six mutation groups when it is present as 5% mutant alleles.

**Table 3:** Analytical sensitivity of formalin-fixed paraffin-embedded tissue DNA blends

**EGFR mutation**	**Mutant specimen No.**	**EGFR nucleic acid sequence**	**Lowest % mutation in the 50 ng/PCR well input to achieve ≥95% “mutation detected” rate (N = 24 replicates)**
**Exon 19 deletion**	1	2235_2249del15	1.39
	2	2236_2250del15	2.53
	3	2238_2252del15	2.37
**L858R**	4	2573 T > G	3.96
	5	2573 T > G	4.19
	6	2573 T > G	4.33
	7	2573 T > G	5.32
**T790M**	7	2369 C > T	2.04
**S768I**	8	2303 G > T	2.42
**Exon 20 insertion**	9	2310_2311insGGT	1.26
**G719X**	10	2156 G > C	2.46
	8	2155 G > T	5.56

**Table 4:** Analytical sensitivity of plasmid DNA blends

**EGFR Mutation**	**Nucleic Acid Sequence**	**Amount of DNA in 5% copy equivalent (ng/25uL) to achieve ≥95% “Mutation Detected” Rate (N = 72 replicates/plasmid)**
**Exon 18 G719A**	2156 G > C	3.13
**Exon 19 Deletion**	2235-2249del15	0.78
**Exon 20 S768I**	2303 G > T	0.78
**Exon 20 T790M**	2369 C > T	3.13
**Exon 20 Insertion**	2307_2308ins9 GCCAGCGTG	3.13
**Exon 21 L858R**	2573 T > G	0.78

### Method correlation and test failure rate

Of the 201 specimens evaluated in the methods correlation between the cobas EGFR test and Sanger sequencing, 49 specimens gave invalid test results for one or both methods produced an invalid result. Forty-eight specimens were invalid by Sanger sequencing (23.8%). Six specimens (3.0%) were invalid by cobas EGFR test using reagent lot 1 (5/6 of these specimens were also invalid by Sanger), and five specimens (2.5%) were invalid using reagent lot 2 (4/5 specimens were also invalid for Sanger).

The comparison of the remaining 152 valid results is shown in Table 5. The OPA between both cobas EGFR test lots and Sanger sequencing was 96.7%, with five discordant specimens for each lot. All specimens yielding discordant resultants with either reagent lot were further analyzed by MPP. Discordant analysis results are listed in Table 6. Sanger sequencing detected two mutation calls (one G719A, one exon 19 deletion) that were not confirmed by the cobas EGFR test or MPP. Two specimens designated “mutation not detected” by Sanger were detected by MPP (exon 19 deletion, exon 20 insertion). Both lots of the cobas EGFR test called one specimen “mutation not detected” that was called as G719S by MPP at 1.1% mutation, which is below the 5% limit of detection of the cobas EGFR test. One specimen was detected as an exon 19 deletion by cobas EGFR lot 2, but not detected for both Sanger and cobas EGFR lot 1. This specimen was detected as an exon 19 deletion at 3% mutation by MPP, which is below the limit of detection of the cobas EGFR test. Lastly, cobas EGFR test lot 1 detected one specimen with an exon 20 insertion. This specimen was called “mutation not detected” by Sanger sequencing, cobas EGFR test lot 2, and MPP.

**Table 5:** Agreement analysis of cobas EGFR mutation test (per lot) versus sanger

		**Sanger**					**Sanger**		
		**MD**	**MND**	**Total**			**MD**	**MND**	**Total**
**cobas EGFR test Lot 1**	**MD**	69	2	71	**cobas EGFR test Lot 2**	**MD**	69	2	71
	**MND**	3	78	81		**MND**	3	78	81
	**Total**	72	80	152		**Total**	72	80	152
Positive agreement = 95.8% (95% CI: 88.3 to 99.1%).					Positive agreement = 95.8% (95% CI: 88.3 to 99.1%).				
Negative agreement = 97.5% (95% CI: 91.3 to 99.7%).					Negative agreement = 97.5% (95% CI: 91.3 to 99.7%).				
Overall agreement = 96.7% (95% CI: 92.5 to 98.9%).					Overall agreement = 96.7% (95% CI: 92.5 to 98.9%).				

CI, confidence interval; MD, mutation detected; MND, mutation not detected.

**Table 6:** Discordant specimen resolution by MPP

**Sample**	**cobas EGFR Test Lot 1**	**cobas EGFR Test Lot 2**	**Sanger**	**MPP**
**1**	MND	MND	G719A	MND
**2**	MND	MND	G719S	G719S (1.1% mutation)
**3**	MND	MND	Exon 19 deletion	MND
**4**	MND	Exon 19 deletion	MND	Exon 19 deletion (3.0% mutation)
**5**	Ex 20 Insertion	Exon 20 insertion	MND	Exon 20 insertion (13.7% mutation)
**6**	Ex 20 Insertion	MND	MND	MND

### Internal Repeatability/External Reproducibility

All runs from the internal repeatability analysis were valid across all specimens, reagent lots, operators, and instruments combined. A single replicate of one specimen gave an invalid result. The specimen was repeated and the valid result replaced the invalid result, which was excluded from data analysis. Initially six (6) false calls out of 192 specimens were observed generating a total percent accuracy of 96.9%. Two of the results were resolved to confirm the observed result by the cobas EGFR test. Three of the false calls were confirmed by MPP; the L858R false call was not confirmed by MPP. With two of the six false calls resolved the assay delivered 188 correct calls out of 192 specimens tested, or an accuracy of 97.9%.

In the external reproducibility study, a total of 2,340 tests were performed on the 13 panel members in 90 valid runs (see Table 2 for list of panel member. No invalid results were obtained. No false positive results were observed, as all 180 replicates of wild-type specimens (95% CI [98–100%]) gave a Mutation Not Detected result. For the exon 19 and exon 21 panel members with 5% mutation, one panel member (EX19_2240_2257del18) had a hit rate below 95% (62.8%, -95% CI [55.3–69.9%]),This may have been due to poor DNA quality in the tumor block used. Although this panel member appeared to have a lower than 95% hit rate, the Ctr SD and CV(%) for this panel member were within the range of the remaining panel members. For all exon 19 and exon 21 panel members with ≤10% mutation had 99.4% (95% CI [96.9–100]) agreement. Overall the external reproducibility study showed little variation in the cobas EGFR test performance at multiple clinical sites (Table 7).

**Table 7:** External reproducibility across reagent lots, operators, instruments, and testing days

**Panel Member**	**Number of Valid Tests**	**Agreement (N)**	**Agreement % (95% CI)**^**a**^
Wild Type	180	180	100 (98.0, 100.0)
EX19_ 2235_2249del15 - 5% Mutation	180	180	100 (98.0, 100.0)
EX19_2236_2250del15 - 5% Mutation	180	180	100 (98.0, 100.0)
EX19_2239_2248 > C - 5% Mutation	180	180	100 (98.0, 100.0)
EX19_2240_2254del15 - 5% Mutation	180	180	100 (98.0, 100.0)
EX19_2240_2257del18 - 5% Mutation	180	113	62.8 (55.3, 69.9)
EX21_ 2573T > G = L858R - 5% Mutation	180	180	100 (98.0, 100.0)
EX19_ 2235_2249del15 - ≤10% Mutation	180	180	100 (98.0, 100.0)
EX19_2236_2250del15 - ≤10% Mutation	180	180	100 (98.0, 100.0)
EX19_2239_2248 > C - ≤10% Mutation	180	180	100 (98.0, 100.0)
EX19_2240_2254del15 - ≤10% Mutation	180	180	100 (98.0, 100.0)
EX19_2240_2257del18 - ≤10% Mutation	180	179	99.4 (96.9, 100.0)
EX21_ 2573T > G = L858R - ≤10% Mutation	180	180	100 (98.0, 100.0)

Note: Results were in agreement when a Mutant Type panel member had a valid result of Mutation Detected or when Wild Type panel member had a valid result of Mutation Not Detected.

^a^ 95% CI = 95% exact binomial confidence interval.

### Interference/Cross-Reactivity/Effects of necrosis

No interference was observed for hemoglobin and triglycerides at CLSI-recommended test concentrations of 2 g/L and 37 mM for any of the 10 FFPET specimens. No interference by therapeutic drugs was observed on the performance of the cobas EGFR test.

No interference from necrotic tissue was observed when evaluating the performance of the cobas EGFR test. Results for all specimens were concordant with Sanger sequencing and MPP results. Thus, levels of necrosis up to 85% did not affect test performance.

Results for the ten FFPET specimens tested under the 13 conditions using the cobas EGFR test matched the expected results for HER2/3/4 cross-reactivity. One specimen that was spiked with the HER4 exon 21 analog plasmid initially produced a result of “Mutation Not Detected”, but yielded the correct call upon retesting. The plasmid with the exon 19 L747S mutation yielded an exon 19 deletion call in all specimens that did not already contain an exon 19 deletion, confirming cross-reactivity between the L747S mutation and the cobas EGFR test. The BLAST (Basic Local Alignment Search Tool) results demonstrated that the primers and probes in the cobas EGFR test are unlikely to cross-hybridize with sequences other than the target sequence. Analogous sequences to the targeted EGFR exons from the HER2, HER3, and HER4 genes did not interfere with the performance of the cobas EGFR test.

### Genotype inclusivity

Results are presented in Additional file 1: Table S1. All of the assessed less common mutations except one (exon 19 deletion mutation 2236_2248 > AGAC) were detected at a similar DNA input level as that for the corresponding predominant mutation. The exon 19 deletion mutation 2236_2248 > AGAC was not consistently detected at any DNA input level.

### Microorganism exclusivity

Neither *Haemophilus influenzae* nor *Streptococcus pneumoniae* had any effect on the performance of the cobas EGFR test (data not shown).

## Discussion

There is a pressing clinical need for a well-validated EGFR testing method with optimal analytical performance, turnaround time, using the least amount of difficult-to-obtain patient specimens. There is also a clear need for guidelines surrounding method performance characteristics. Here, we present results on seven out of 25 analytical validation studies performed on over 200 clinical FFPET specimens as well as external reproducibility study of the test run at multiple clinical sites. It is important to note that validation studies were performed on plasmid specimens as well as FFPET specimens, allowing an accurate understanding the of test performance in typical clinical specimens. Performance of the test in alternative specimen types is currently being conducted.

One commonly used method for interrogating mutations in the EGFR gene is Sanger sequencing. Sanger sequencing is highly variable based on lab-validated protocols. In some cases, Sanger sequencing takes up to 600 ng of DNA to interrogate all 4 exons in the EGFR gene [16]. Particularly in the field of NSCLC, where patient samples are difficult to obtain and testing (molecular and immunohistochemical) is being prioritized for treatment decisions, the efficient use of limited specimen is of great importance. The cobas EGFR test detects 41 mutations in exons 18, 19, 20, and 21 and uses 150ng of total DNA input. The studies described in this manuscript indicate that the cobas EGFR test is able to detect mutations in EGFR exons 18, 19, 20, and 21 at ≥5% mutation level using only 50 ng of DNA per reaction well, an amount that typically can be extracted from a single 5 μm curl. The cobas EGFR test was able to detect mutations that were confirmed by MPP but not detected by Sanger sequencing. The increased sensitivity of the cobas EGFR test is consistent with previous studies of other PCR-based mutation assays [17-19]. The sensitivity of Sanger sequencing may be increased to some extent by taking measures to enrich for tumor tissue, such as macrodissection or laser microcapture. However, these measures require extra time and effort on the part of the pathologist, and in some cases require the use of specialized equipment. By contrast, the cobas EGFR test does not require macrodissection unless the estimated tumor content in the specimen is below 10%.

To confirm the greater sensitivity of the cobas EGFR test compared to Sanger, a third comparator method was used, MPP. To eliminate any sequencing bias, both Sanger sequencing and MPP were performed by an external laboratory that was blinded to the results of the cobas EGFR test. In the four of six cases, MPP confirmed the cobas EGFR test result. The Sanger sequencing provided two false positive mutation calls and two false negative mutation calls, which in the clinical setting would have resulted in two patients who would be unlikely to respond to treatment, receiving treatment, and patients who would benefit from treatment being denied the intervention. Occasional false positive results with Sanger sequencing have been observed in other studies [17,20,21], perhaps reflecting some inherent subjectivity in the interpretation of Sanger sequencing results. Such subjectivity is eliminated from the cobas EGFR test, as the analysis and reporting of results are fully automated.

Low invalid rates expedite time to result and avoiding the unnecessary use of additional specimens for retesting. Of interest, the low invalid rates were observed despite the samples being between 3 and 10 years old. The studies also show that the cobas EGFR test is more robust than Sanger sequencing with a lower invalid test rate (3% for cobas vs 23.8% for Sanger). Very few reported method comparison studies have compared invalid test rates between different assay methods. However, we have previously demonstrated very low invalid test rates for other mutation assays on this platform [17,20].

A further benefit of the cobas EGFR test is its rapid turnaround time (~1 day for 24 samples; 1 kit), which is considerably shorter than for Sanger sequencing (~5 days). The slower turnaround time for Sanger sequencing and its higher invalid test rate, which potentially results in the need for reanalysis, could lead to important delays in patients receiving appropriate treatment for NSCLC. This is an important concern as the majority of patients present with advanced, disseminated disease [22]. This rapid and sensitive method enables efficient testing of limited tissue specimens, where patient samples are difficult to obtain and molecular testing must be prioritized for treatment algorithms.

As part of the validation of the cobas EGFR test we examined both internal repeatability and external reproducibility. In the internal repeatability analysis, the cobas EGFR test had high accuracy (98%) across all specimens, reagent lots, operators, and instruments combined. High reproducibility was observed in the external reproducibility analysis although one sample was observed to contribute a disproportionate amount to the variability observed. This sample had 5% mutation; however, analysis at ≤10% improved reproducibility to >97%. An evaluation of *EGFR* testing in 15 French centers showed low concordance between sites, ranging from median kappa values of 0.47 (0.45-0.49) for Exon 19 and 21, underpinning the critical need to set standards for *EGFR* mutation testing [8,23]. The external reproducibility study is targeted for submission alongside results from clinical trial entitled, “Phase III Study (Tarceva®) vs Chemotherapy to Treat Advanced Non-Small Cell Lung Cancer (NSCLC) in Patients With Mutations in the TK Domain of EGFR” (clinical trial # NCT00446225). The clinical utility of the cobas EGFR test was assessed through a retrospective analysis of specimens from the EURTAC trial (clinical trial # NCT00446225). Though there has been consideration of the use of next generation sequencing in routine clinical diagnostics, for the accurate selection of patient therapy, method of testing for EGFR mutations should be well validated both clinically and analytically.

Our study also demonstrated that a variety of potential interfering substances – including endogenous substances, common medications, and respiratory microorganisms – had no significant effect on the assay’s analytic performance. A thorough understanding of the specimen attributes that could affect a molecular assay are a key component of test optimization and validation.

## Conclusions

The analytic studies presented here show that the cobas EGFR test is a sensitive, accurate, rapid, and reproducible assay for *EGFR* mutations that allows clinicians to identify those patients with advanced NSCLC who have a high likelihood of benefiting from treatment with anti-EGFR TKI therapies.

## Abbreviations

EGFR: Epidermal growth factor receptor; NSCLC: Non-small cell lung cancer; FFPET: Formalin-fixed paraffin-embedded tissue; MPP: Massively parallel pyrosequencing; OPA: Overall percent agreement; NPA: Negative agreement; PPA: Positive agreement; TKI: Tyrosine kinase inhibitors; AS-PCR: Allele-specific polymerase chain reaction.

## Competing interests

All authors except KB, SA, and WM are employees of Roche Molecular Systems. HJL is a former employee for RMS. Kits and specimens were provided by RMS for the clinical reproducibility study.

## Authors’ contributions

PA, JF, JS, RC, TR, JT, HBT, SC, and MC contributed to study design and running all analytical performance and verification testing. FS was involved in drafting the manuscript and interpretation of the data. WW, LU, SS were involved in study design and acquisition of the data. HJL oversaw the study design and conduct of the external reproducibility study and was involved in drafting of the manuscript. RS was involved in the study design and conduct of the clinical reproducibility study. KB, WM, and SA performed all clinical reproducibility studies and data analysis. All authors have read and approved the final version of the manuscript.

## Pre-publication history

The pre-publication history for this paper can be accessed here:

http://www.biomedcentral.com/1471-2407/13/210/prepub

## Supplementary Material

Additional file 1: Table S1 Genotype inclusivity at minimum or target detection for rare EGFR mutations. Click here for file
