# Supplementary material for: Analytic performance studies and clinical reproducibility of a real-time PCR assay for the detection of epidermal growth factor receptor gene mutations in formalin-fixed paraffin-embedded tissue specimens of non-small cell lung cancer
Source: BMC Cancer. 2013 Apr 27;13:210. doi: 10.1186/1471-2407-13-210 (PMC3660201; doi:10.1186/1471-2407-13-210)
Supplement: Additional file 1: Table S1 — Genotype inclusivity at minimum or target detection for rare EGFR mutations. [file 1471-2407-13-210-S1.doc]

**Table S1.** Genotype inclusivity at minimum or target detection for rare EGFR mutations

| **Exon** | **Nucleic acid change** | **Amino acid change** | **Cosmic ID** | **Detection** |
| --- | --- | --- | --- | --- |
| **Exon 18** | 2155G>A | G719S | 6252 | Yes |
|  | 2155G>T | G719C | 6253 | Yes |
| **Exon 19** | 2236_2250del15 | E746-A750del | 6225 | Yes |
|  | 2240_2257del18 | L747-P753>S | 12370 | Yes |
|  | 2240_2254del15* | L747-T751del | 12369 | Yes |
|  | 2239_2256del18 | L747-S752del | 6255 | Yes |
|  | 2239_2251>C | L747-T751>P | 12383 | Yes |
|  | 2237_2251del15 | E746-T751>A | 12678 | Yes |
|  | 2237_2255>T | E746-S752>V | 12384 | Yes |
|  | 2239_2248TTAAGAGAAG>C | E747-A750>P | 12382 | Yes |
|  | 2239_2253del15* | L747-T751del | 6254 | Yes |
|  | 2239_2247del9 | L747-E749del | 6218 | Yes |
|  | 2235_2252>AAT | E746_T751>I | 13551 | Yes |
|  | 2236_2253del18 | E746_T751del | 12728 | Yes |
|  | 2237_2254del18 | E746_S752>A | 12367 | Yes |
|  | 2238_2255del18 | E746_S752>D | 6220 | Yes |
|  | 2238_2248>GC | L747_A750>P | 12422 | Yes |
|  | 2238_2252>GCA | L747_T751>Q | 12419 | Yes |
|  | 2239_2258>CA | L747_P753>Q | 12387 | Yes |
|  | 2240_2251del12 | L747_T751>S | 6210 | Yes |
|  | 2233_2247del15 | K745_E749del | 26038 | Yes |
|  | 2253_2276del24 | S752_I759del | 13556 | Yes |
|  | 2235_2248>AATTC | E746_A750>IP | 13550 | Yes |
|  | 2237_2252>T | E746_T751>V | 12386 | Yes |
|  | 2235_2251>AATTC | E746_T751>IP | 13552 | Yes |
|  | 2235_2255>AAT | E746_S752>I | 12385 | Yes |
|  | 2237_2253>TTGCT | E746_T751>VA | 12416 | Yes |
|  | 2237_2257>TCT | E746_P753>VS | 18427 | Yes |
|  | 2238_2252del15 | L747_T751del | 23571 | Yes |
|  | 2239_2256>CAA | L747_S752>Q | 12403 | Yes |
|  | 2236_2248>AGAC | E746_A750>RP | 12413 | Not Detected |
| **Exon 20** | 2319_2320insCAC | H773_V774insH | 12377 | Yes |
|  | 2310_2311insGGT | D770_N771insG | 12378 | Yes |
|  | 2309_2310(AC>CCAGCGTGGAT) | V769_D770insASV | 13558 | Yes |
|  | 2311_2312ins9(GCGTGGACA) | D770_N771insSVD | 13428 | Yes |
| **Exon 21** | 2573_2574TG>GT | L858R | 12429 | Yes |
